# Association Between Unsaturated Fatty Acid Levels and Chronic Obstructive Pulmonary Disease: A Bidirectional Mendelian Randomization Study

**DOI:** 10.1111/crj.70179

**Published:** 2026-03-07

**Authors:** Shuai Jiang, Lili Lu, Xiaojun Wang, Xunxia Zhu, Xiaoyu Chen, Hung‐Chen Chang, Xuchao Gu, Fuzhi Yang, Xuanqi Liu, Xuelin Zhang, Xiaoyong Shen

**Affiliations:** ^1^ Department of Thoracic Surgery Huadong Hospital Affiliated to Fudan University Shanghai China; ^2^ Department of Traditional Chinese Medicine Huadong Hospital Affiliated to Fudan University Shanghai China; ^3^ Shanghai Key Laboratory of Clinical Geriatric Medicine Huadong Hospital Affiliated to Fudan University Shanghai China; ^4^ Department of Pulmonary and Critical Care Medicine Zhongshan Hospital, Fudan University Shanghai Medical College Shanghai China

**Keywords:** chronic obstructive pulmonary disease (COPD), Mendelian randomization (MR), risk factors, unsaturated fatty acids (UFAs)

## Abstract

**Background:**

Observational studies have revealed that the levels of unsaturated fatty acids (UFAs) may influence the development, progression, and management of chronic obstructive pulmonary disease (COPD). To investigate the association between UFAs and COPD, we performed a bidirectional Mendelian randomization (MR) study.

**Methods:**

We extracted summary genome‐wide association statistics (GWAS) for UFAs (*N* = 115 082) among population from UK Biobank study by measuring circulating lipoprotein lipid concentrations. The genetic instrument for COPD was derived from the FinnGen, which included 338 303 COPD controls and 20 066 cases of the disease. Measured at the genome‐wide significance level, independent genetic variations associated with each characteristic were considered instrumental factors. Two‐sample MR analysis was mainly conducted utilizing the inverse‐variance‐weighted (IVW) approach, complemented by the weighted median method and the MR‐Egger regression.

**Results:**

IVW MR analysis significantly demonstrated that the level of docosahexaenoic acid (DHA) (OR 0.812, 95% CI 0.719–0.918, *p* < 0.001), linoleic acid (LA) (OR 0.850, 95% CI 0.781–0.926, *p* < 0.001), the levels of omega‐3 fatty acids (OR 0.884, 95% CI 0.781–0.99, *p* = 0.049), omega‐6 fatty acids (OR 0.878, 95% CI 0.812–0.950, *p* = 0.001), and polyunsaturated fatty acids (PUFAs) (OR 0.901, 95% CI 0.827–0.982, *p* = 0.018) all linked to a higher risk of COPD. Moreover, in reverse direction MR analysis, genetic liability to COPD showed associations with higher levels of monounsaturated fatty acids (MUFAs) (OR 1.040, 95% CI 1.010–1.071, *p* = 0.008). Horizontal pleiotropy is not likely to materially skew the causative estimates from sensitivity analysis.

**Conclusion:**

Our research added credence to the current evidence that suggests a bidirectional causal link between UFAs and COPD. It is imperative to comprehend this connection in order to effectively prevent and manage COPD.

## Graphical abstract

Our MR analysis demonstrated that kinds of unsaturated fatty acids (UFAs) could be causal protective factors of chronic obstructive pulmonary disease (COPD). It reminded people to modify dietary nutrition, especially increasing the intake of UFAs to prevent COPD.

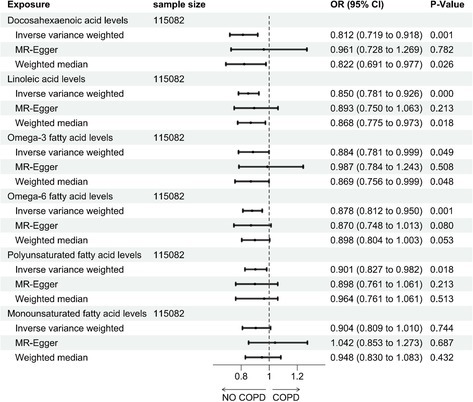

**Abbreviations**
COPD: chronic obstructive pulmonary disease
COX: cyclooxygenase
CRP: C‐reactive protein
CYP: cytochrome P450 mixed function oxidase enzymes
DHA: docosahexaenoic acid
ECM: extracellular matrix
FAs: fatty acids
FOXO: forkhead box
GWAS: genome‐wide association statistics
IL: interleukin
IVs: instrumental variables
IVW: inverse‐variance weighted
Keap1: Kelch‐like ECH‐associated protein 1
LA: linoleic acid
LOX: lipoxygenase
MR: Mendelian randomization
MUFAs: monounsaturated fatty acids
Nrf2: negative regulator of the nuclear factor erythroid 2–related factor
PM2.5: particulate matter with diameter < 2.5 μm
PUFAs: polyunsaturated fatty acids
RCT: randomized controlled trial
SCD1: stearoyl‐coenzyme A desaturase 1
SFAs: saturated fatty acids
SIRT1: sirtuin1
SNP: single nucleotide polymorphism
SOC: superoxide dismutase
TNF: tumor necrosis factor
UFAs: unsaturated fatty acids
UKB: UK Biobank

## 1 Introduction

Chronic obstructive pulmonary disease (COPD) is a main source of healthcare usage and ranks third among the leading causes of death globally [1, 2]. The prevalence of COPD worldwide in 2019 was 10.6% among individuals aged 30–79 years [3]. COPD is characterized by irreversible airflow obstruction due to emphysema, alveolar destruction, and inflammation [4]. It is anticipated that the burden of COPD will keep increasing in the ensuing decades due to the aging of the world population.

Diet and nutrition may represent crucial modifiable risk factors influencing the development, progression, and management of COPD [5, 6, 7]. Fatty acids (FAs) are essential nutrients for the human body [8, 9]. Generally speaking, saturated fatty acids (SFAs) are considered to be prevalent risk factors for various diseases, such as cardiovascular disease [10], fatty liver, and diabetic nephropathy [11, 12], whereas unsaturated fatty acids (UFAs) are generally regarded as protective factors [13, 14, 15, 16]. The latter can be classified into polyunsaturated fatty acids (PUFAs) and monounsaturated fatty acids (MUFAs) [17]. FAs with more than one double bond on their carbon chain are known as PUFAs [18]. They are categorized as omega‐6 or omega‐3 relying on where the initial double bond is located in relation to the methyl end. These two families each encompass FAs with varying degrees of unsaturation and carbon chain lengths [19]. The current consensus is that the majority of lipid mediators regulating inflammation are metabolites originating from omega‐3 and omega‐6 FAs [20]. Omega‐3 FAs have been demonstrated to have a positive influence on blood triglyceride levels, blood pressure, fasting blood glucose, insulin resistance, and high‐density lipoprotein cholesterol, likely clarifying their protective effects against cardiovascular disease [21]. Likewise, omega‐6 PUFAs can also benefit blood lipid management, particularly low‐density lipoprotein cholesterol, as well as insulin resistance [22]. PUFAs undergo three main enzymatic pathways to produce oxylipin, as lipoxygenase (LOX), cyclooxygenase (COX), and cytochrome P450 mixed function oxidase enzymes (CYP). These pathways significantly increase their range of biological functions [23, 24]. Until now, the knowledge regarding UFAs in the field of respiratory diseases needs further deep investigation.

Previous investigations have displayed that in COPD patients, high omega‐3 FA intake was profitable and associated with fewer severe exacerbations in the previous 3 months and an increase in lung function [25, 26], relying on its anti‐inflammatory effects [13]. Meanwhile, higher level of omega‐6 FAs was linked to lower FEV1, and high value of omega‐6 to omega‐3 FAs was associated with bad outcomes in COPD patients [25]. Omega‐6 FAs are predominantly described to promote inflammation; nevertheless, omega‐3 FAs are generally excogitated as inhibiting inflammation [20]. However, several studies showed no significant connection between omega‐3 FA intake with prevalence combined with outcome of COPD [27, 28].

With the fast progress of genomics, more and more pieces of evidence are emerging and highlighting the impact of heritability on disease etiology. Mendelian randomization (MR) as an effective genetic epidemiological approach has received increasing attention [29]. MR investigations are less vulnerable to environmental confounding variables than observational research because genetic variations are established at the time of conception [29, 30]. The two‐sample MR approach enhances statistical capacity for estimating causal associations between exposures and outcomes by leveraging available summary estimates from various huge massive genome‐wide association statistics (GWAS) datasets [31]. In this research, we conducted an extensive and thorough MR study to explore the probable causal‐and‐effect associations among UFAs and COPD. This two‐sample MR method might help screen the underlying risk factors of COPD and evaluate its biologic value.

## 2 Methods

### 2.1 Study Design

**FIGURE 1:**
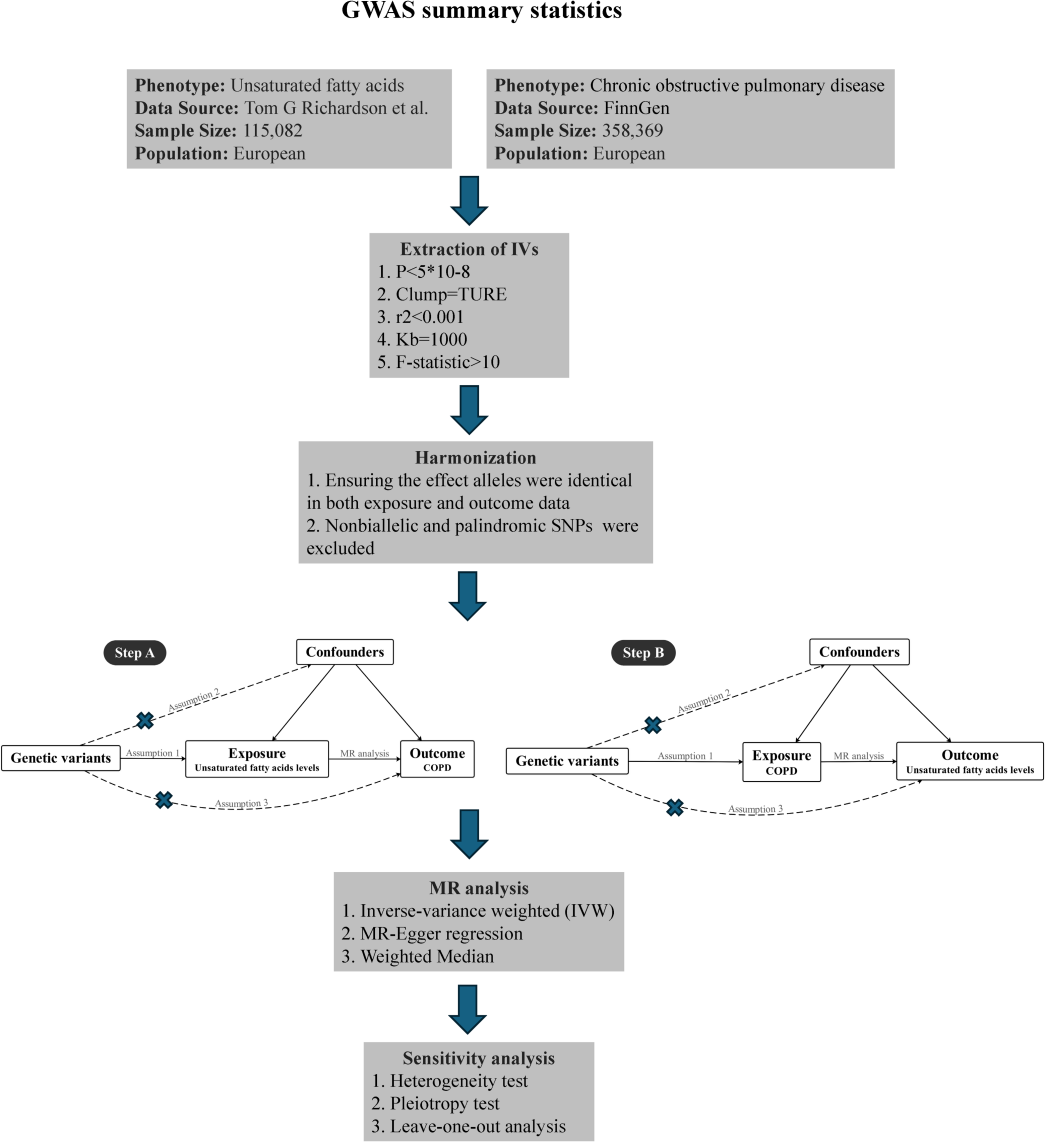
The flowchart of causal inference between UFAs and COPD. GWAS, genome‐wide association statistics; IVW, inverse‐variance‐weighted; MR, Mendelian randomization; SNP, genome‐wide association statistics.

As illustrated in Figure 1, our study consisted of two main components and was structured as a bidirectional two‐sample MR research: Step A: Investigation of causal relationships of UFAs on COPD. Step B: Investigation of causal relationships of COPD on UFAs. MR relies on three fundamentals to assure the causal inference was valid: (1) The exposure factors and instrumental variables (IVs) have a substantial relationship. (2) The assumption of independence implies that IVs should not exhibit associations with confounding variables. (3) IVs only affect the result by means of exposure; they do not directly affect the outcome [32, 33].

### 2.2 Data Source

It is necessary to obtain GWAS summary of exposure factors and results from different databases for MR analysis. For the COPD, European ancestry summary‐level GWAS statistics were acquired from the FinnGen (Freeze 10) (https://www.finngen.fi/en) [34]. The COPD definition depends on ICD codes. These data contain 20 066 cases and 338 303 controls. The FinnGen investigators used mixed‐model logistic regression to modify the findings for age, gender, 10 primary elements, and genotyping batch [34].

As to UFAs, GWAS data were acquired from IEU Open GWAS website (https://gwas.mrcieu.ac.uk/). These data contain 115 082 European ancestry individuals from the UK Biobank study, characterizing circulating lipoprotein lipid concentrations through drug–target MR [35]. The study examined the genetically anticipated impacts of lipid‐modifying therapies on the human metabolic profile.

### 2.3 Genetic Instrument Selection

To provide unbiased estimates of the effect of exposures on outcomes, the choice of genetic tools must obey these three basic presumptions mentioned above [36]. Firstly, we obtained single nucleotide polymorphisms (SNPs) of UFAs as instruments, which are connected to UFAs within a genome‐wide significance criterion of *p* < 5 × 10^−8^. Secondly, we utilized the residual SNPs at a threshold of linkage disequilibrium clumping *r* ^2^ < 0.001 surpassing a 10 Mb window using the European sample of 1000 genome data as a comparison panel to avoid collinearity within SNPs. Thirdly, we scanned these SNPs in PhenoScanner (www.phenoscanner.medschl.cam.ac.uk) and eliminated SNPs related to confounders or outcomes. Confounder primarily denoted the prospective risk variables apart from the exposure of interest that might lead to the result. Typical factors contributing to COPD contained but were not restricted to aging, obesity, asthma, tobacco use, air pollution, and respiratory infections [37, 38]. In the next step, SNPs were harmonized to guarantee that the effect alleles were the same between exposure and outcome data. Palindromic and nonbiallelic SNPs (A/T and G/C) were not included [39]. Lastly, we computed the *F*‐statistic value of each SNP to estimate its power. SNPs with *F*‐statistic values exceeding 10 suggested robust instruments that may prevent bias from weak instruments [40]. This is important in genetic studies, as using weak instruments can lead to biased estimates and inaccurate conclusions. The *F*‐statistic values of all the SNPs in our MR analysis were greater than 10. We archived all harmonized SNPs for each exposure–outcome pair (Table S1).

### 2.4 MR Analysis and Sensitivity Analysis

For the MR study, the primary analysis method used to establish a cause‐and‐effect relationship between COPD and UFAs was the inverse‐variance weighted (IVW) approach [41]. Horizontal pleiotropy can be objectively estimated by IVW [42]. To evaluate causal association, we conducted the weighted median model and MR‐Egger regression model. Notably, the weighted median method provides dependable estimates, especially when over 50% of the information is derived from valid IVs [43]. Meanwhile, the IVW approach and the MR‐Egger method are employed in estimating the heterogeneity of the chosen IVs [44].

In our investigation, Cochran's *Q* was performed to quantify heterogeneity, with *p* < 0.05 suggesting solid and reliable results [45]. Additionally, horizontal pleiotropy was assessed utilizing MR‐Egger regression intercept testing, where a nonzero intercept would suggest the existence of horizontal pleiotropy [41]. Ultimately, leave‐one‐out analysis was employed to verify that our MR results were not disproportionately impacted by individual SNPs. All statistical analyses were conducted utilizing the “TwoSampleMR” packages in R software Version 4.3.2.

## 3 Results

### 3.1 IVs

Basic information of the contributing GWAS is summarized in Figure 1. We excluded the SNPs connected with the outcome or probable confounders after identifying these IVs in the PhenoScanner database. In MR analysis with UFAs as exposures, 25 SNPs were decided to genetically predict DHA levels, and 36 SNPs were selected to predict LA levels totally. Meanwhile, we chose 34 SNPs to predict omega‐3 FA levels, as well as 45 SNPs for omega‐6 FA levels. As to PUFA and MUFA levels, their predictions were based on 40 and 43 SNPs. In MR analysis with COPD as exposure, we genetically identified 17 SNPs as IVs. The *F*‐statistics of these IVs are more than 10 after computation.

### 3.2 The Causal Influence of UFAs on COPD Risk

**FIGURE 2:**
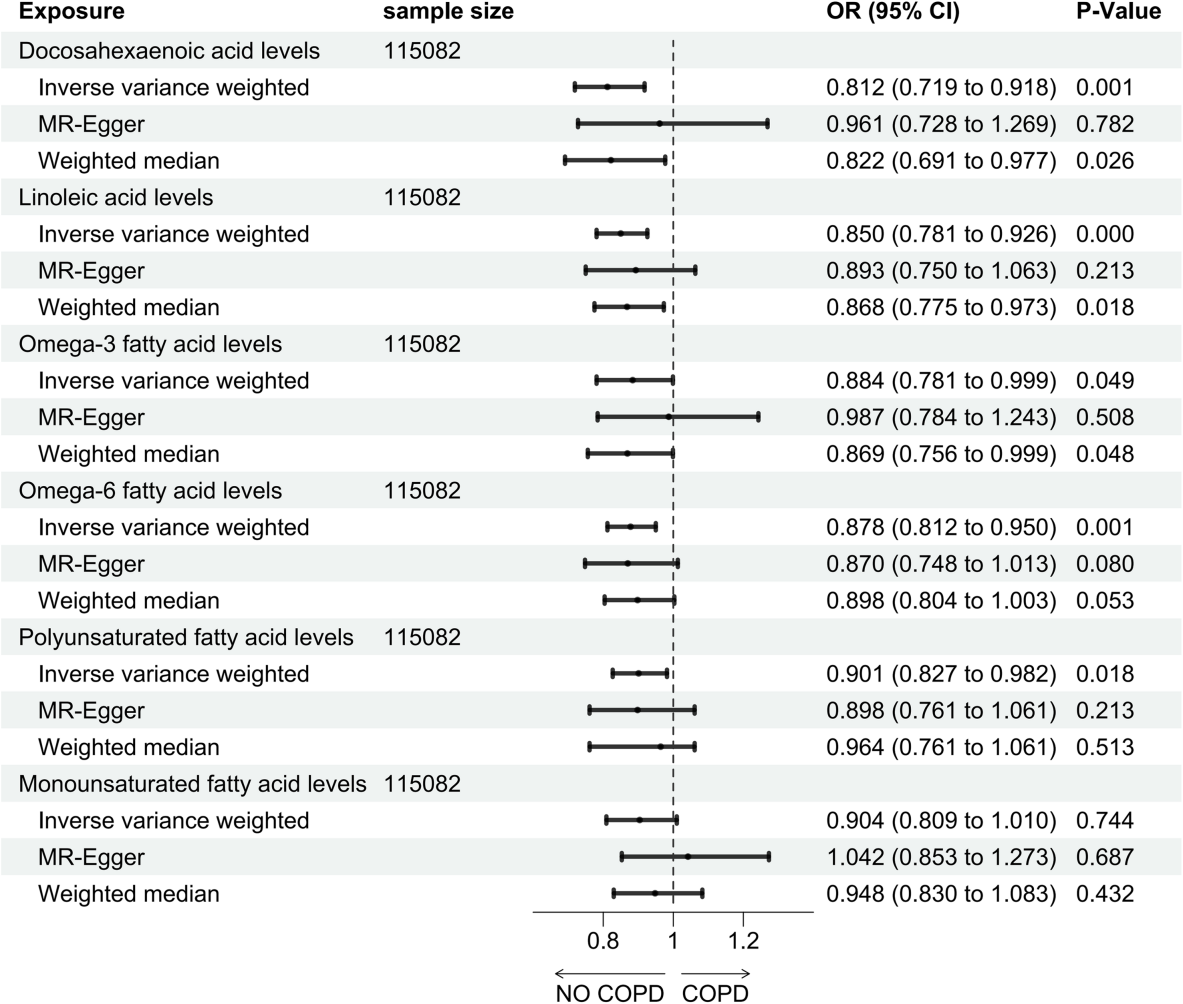
MR results of effect of UFA levels on COPD.

The consequences from the MR study of UFAs are exhibited in Figure 2. In the discovery phase, the IVW method and weighted median method genetically demonstrated that higher levels of DHA decreased the risk of COPD (IVW: OR 0.812, 95% CI 0.719–0.918, *p* < 0.001; weighted median: OR 0.822, 95% CI 0.691–0.977, *p* = 0.026). Nevertheless, the association was not corroborated with the result of MR‐Egger (MR‐Egger: OR 0.961, 95% CI 0.782–1.269, *p* = 0.782) (Figure 2). Notably, IVW and weighted median evaluations of 36 valid IVs' effects for LA on COPD provided proof to support that a causal association existed (IVW: OR 0.850, 95% CI 0.781–0.926, *p* < 0.001; MR‐Egger: OR 0.893, 95% CI 0.750–1.063, *p* = 0.213; weighted median: OR 0.868, 95% CI 0.775–0.973, *p* = 0.018), suggesting that a high level of LA could also decrease the risk of suffering from COPD by 10.7% (Figure 2).

As to omega‐3 FA levels, a weak positive effect was observed on COPD (IVW: OR 0.884, 95% CI 0.781–0.999, *p* = 0.049; MR‐Egger: OR 0.987, 95% CI 0.784–1.243, *p* = 0.508; weighted median: OR 0.869, 95% CI 0.756–0.999, *p* = 0.048). Simultaneously, the positive causal inference of omega‐6 FAs on COPD was observed in the IVW method (IVW: OR 0.878, 95% CI 0.812–0.950, *p* = 0.001), indicating that individuals with elevated levels of omega‐6 FAs experienced a 7.8% reduced likelihood of developing COPD. However, this was negative in MR‐Egger and weighted median methods (MR‐Egger: OR 0.870, 95% CI 0.748–1.013, *p* = 0.080; weighted median: OR 0.898, 95% CI 0.804–1.003, *p* = 0.053). It is worth noting that PUFAs are protective factors for COPD (IVW: OR 0.901, 95% CI 0.827–0.982, *p* = 0.018), which means people with a high level of PUFAs may have approximately 10% lower probability of suffering from COPD. At last, our study exhibited no prospective connection between MUFAs levels and COPD (IVW: OR 0.904, 95% CI 0.809–1.010, *p* = 0.744) (Figure 2).

### 3.3 Effect of COPD on UFA Levels

**FIGURE 3:**
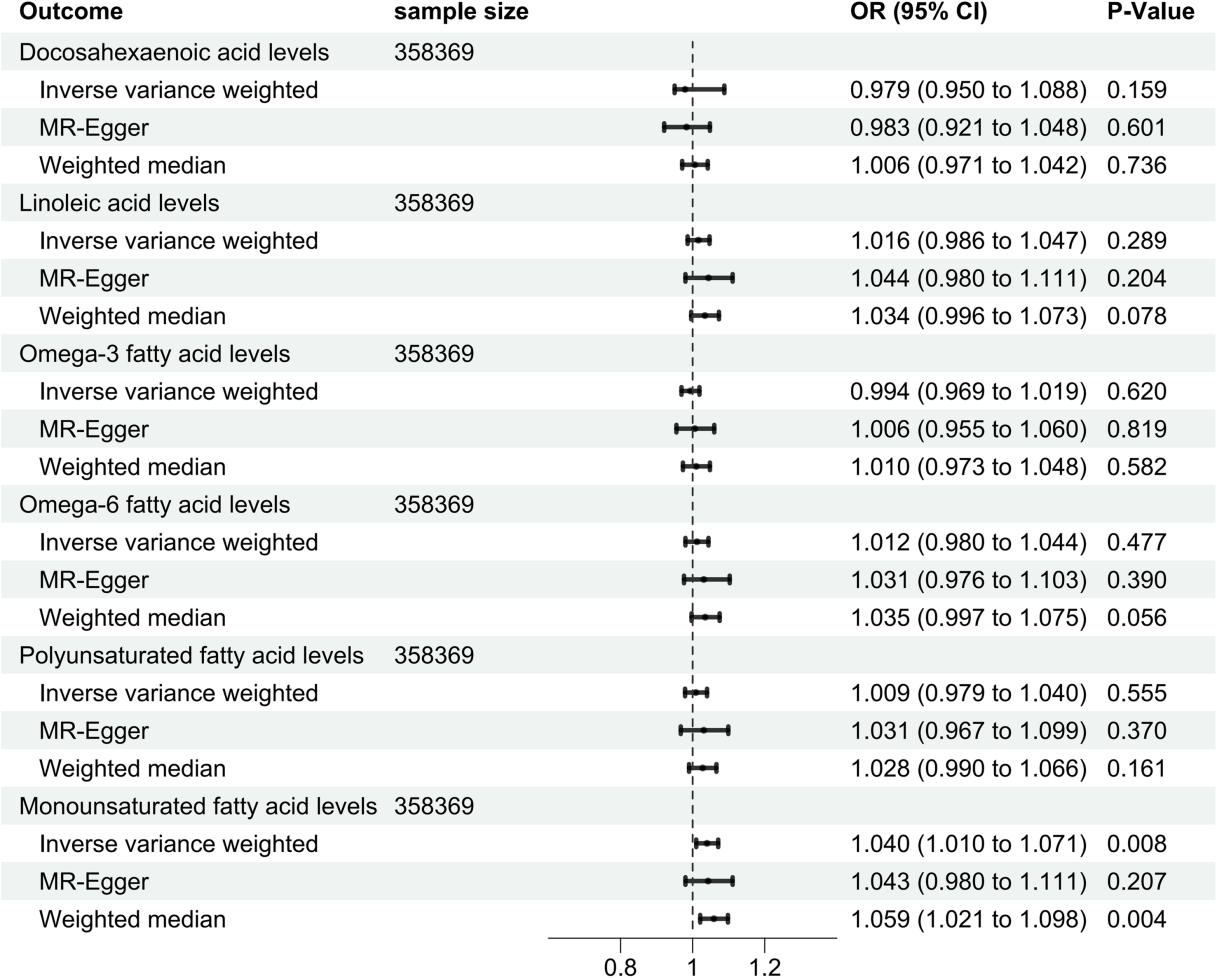
MR results of effect of COPD on UFA levels.

Furthermore, we conducted reverse MR investigation to explore the possibility of them. There was no proof from the reverse MR analysis that COPD affected PUFAs. As is shown in Figure 3, no evidence was found for COPD affecting the levels of DHA, LA, omega‐3 FAs, omega‐6 FAs, and PUFAs (*p* > 0.05). Interestingly, there was a significant effect of COPD on MUFAs (IVW: OR 1.040, 95% CI 1.010–1.071, *p* = 0.008; MR‐Egger: OR 1.043, 95% CI 0.980–1.111, *p* = 0.207; weighted median: OR 1.059, 95% CI 1.021–1.098, *p* = 0.004) (Figure 3), indicating that COPD might increase the level of MUFAs.

### 3.4 Sensitivity Analysis

**FIGURE 4:**
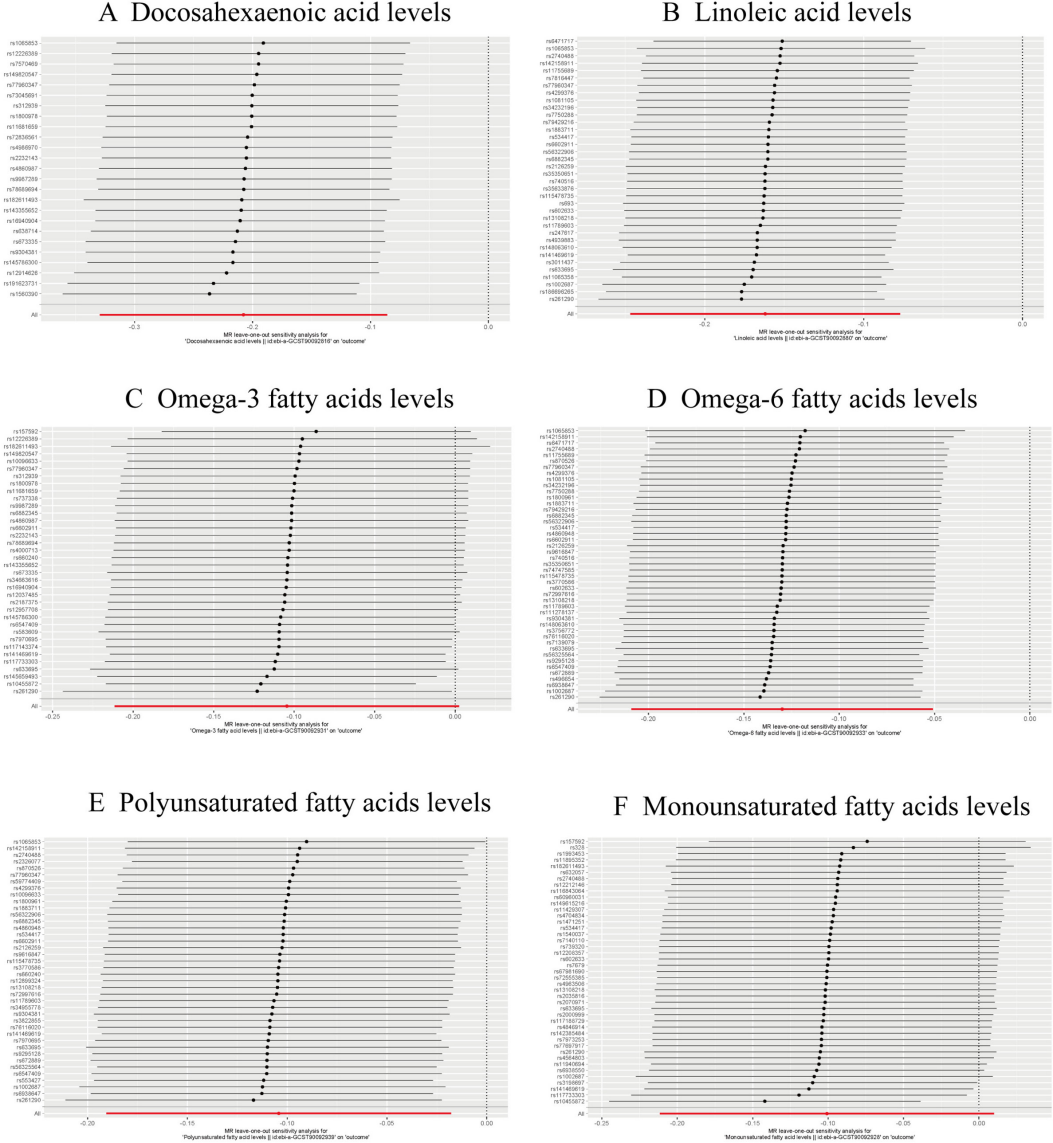
Leave‐one‐out sensitivity tests to assess possible horizontal pleiotropy. (A) Docosahexaenoic acid levels. (B) Linoleic acid levels. (C) Omega‐3 fatty acid levels. (D) Omega‐6 fatty acid levels. (E) Polyunsaturated fatty acid levels. (F) Monounsaturated fatty acid levels.

The sensitivity analysis yielded consistent results overall. Other than the impact of omega‐3 FA levels and MUFA levels on COPD, Cochran's *Q* test indicated that no substantial heterogeneity within the chosen SNPs (*p* > 0.05). The MR‐Egger intercept analysis revealed no significant deviation from zero for any analysis (all *p* > 0.05), suggesting the absence of horizontal pleiotropy (all *p* > 0.05). We additionally utilized the leave‐one‐out method to check for possible horizontal pleiotropy and observed no indications of confounding caused by pleiotropy among SNPs (all *p* > 0.05) (Figure 4). The aforementioned findings suggested that this analysis had adequate statistical capability and the MR findings held well under sensitivity analysis.

## 4 Discussion

In recent years, the bioactive functions of the gut–lung axis have gradually raised the attention of researchers [46]. The term “gut–lung axis” describes the bidirectional communication and exchange of information between the gastrointestinal system (gut) and lung. It involves the exchange of signals, immune cells, and metabolites including FAs between these two organs, which can influence both gut and lung health [47, 48]. The gut–lung axis is particularly relevant in the context of COPD, a respiratory condition associated with changes in intestinal health [49]. Dietary factors, such as PUFAs, have been implicated in the gut–lung axis. PUFAs, particularly omega‐3 FAs, can alter neutrophil chemotaxis, eicosanoid production, and the composition of lipid membranes, which are potential modulators of lung disease [50, 51]. There seems to be a delicate relationship between the content of UFAs in the human body and COPD. Nonetheless, there is a lack of suitable randomized controlled trials (RCTs) that can effectively uncover these causal associations at present.

Based on our extensive research, this is the initial analysis utilizing MR to excogitate the causal connection between UFAs and COPD. We discovered that not only are UFAs impactful on COPD, but COPD also exerts an influence on MUFAs levels, and these associations were robust to sensitivity analysis. The results showed that levels of DHA, LA, omega‐3, omega‐6, and PUFAs are all protective on COPD. DHA is the most prevalent omega‐3 FAs [52], and LA falls into the category of omega‐6 FAs, and both omega‐3 and omega‐6 FAs are classified as PUFAs [53]. These findings are consistent with the consensus that UFAs are associated with the progress of COPD [25, 26, 54]. Ahmadi et al. observed the lower omega‐3 FA intake in dietary habits of COPD patients than normal population [26]. Shahar et al. also found that higher total omega‐3 FA consumption was strongly connected with lower odds for COPD; in other words, omega‐3 FAs are protective factors on COPD and deterioration of lung function [54], which was consistent with our results.

According to previous studies, PUFAs, especially omega‐3 FAs, are protective factors for COPD owing to their anti‐inflammatory consequence, which is consistent with our MR study [55]. In a randomized clinical trial involving 46 COPD patients, Engelen et al. investigated omega‐3 FA supplementation effects on functional status and metabolism. The researchers observed significantly reduced levels of several pro‐inflammatory cytokines—including interleukin‐2 (IL‐2), IL‐17, IL‐6, and tumor necrosis factor‐β (TNF‐β)—in the supplementation group compared to the control group [56]. Omega‐3 FAs played a significant role in cellular mechanisms involved in integration into cellular membranes and subsequent alteration of eicosanoid synthesis to possess anti‐inflammatory qualities [57]. Previous experimental studies also revealed that long‐chain omega‐3 FAs reduced the production of pro‐inflammatory PGE2 and LTB4 by inflammatory cells while also inhibiting the activity of NF‐κB [58] and downregulating the release of cytokines generated from monocytes and macrophages, including IL‐1β and TNF‐α [59]. Although omega‐6 FAs have predominantly been correlated with pro‐inflammatory effects [20], a study demonstrated that omega‐6 FAs had a more significantly inhibitory impact on the basal extracellular matrix (ECM)–protein expression and deposition in COPD cells [60]. This may partially explain why omega‐6 FAs were protective factors in our MR study.

Oxidative stress is another major driving mechanism in the pathogenesis of COPD [61]. In fact, omega‐3 FAs may act as antioxidants by regulating the signaling pathway [21]. Oxidized omega‐3 FAs target Kelch‐like ECH‐associated protein 1 (Keap1), which is a negative regulator of the nuclear factor erythroid 2–related factor 2 (Nrf2). By interacting with Keap1 and prompting the release of Nrf2, oxidized omega‐3 FAs promote the production of antioxidant genes [62]. Additionally, oxidized omega‐3 FAs perform on forkhead box (FOXO) and sirtuin1 (SIRT1) proteins, ultimately leading to an increase in superoxide dismutase (SOD) levels. This enhancement of SOD helps to defend cells from oxidative damage, highlighting the potential protective influences of omega‐3 FAs in combating oxidative stress associated with COPD [63]. However, the role of PUFAs in the protease–antiprotease imbalance mechanism has not yet been identified.

The findings above indicate that dietary or clinical interventions may exert an impact on the onset and progression of COPD. In fact, several studies have put the regulation of FAs into practice. de Batlle et al. provided the first evidence linking dietary intake of omega‐3 and omega‐6 FAs to serum inflammatory markers in patients with COPD. Their cross‐sectional analysis included 250 patients with COPD in a clinically stable state. They found that increased omega‐3 intake was associated with reduced TNF‐α levels, whereas high omega‐6 intake correlated with elevated C‐reactive protein (CRP) and IL‐6 concentrations [64]. Recently, another review also indicated that implementing preventive, standardized, and personalized dietary interventions could ameliorate intestinal dysbiosis in COPD patients. This enhancement promotes the production of short‐chain FAs, thereby reducing inflammation and slowing COPD progression. Moreover, adequate intake of specific FAs may further improve COPD outcomes [65]. Nevertheless, clinical trials in this area remain limited and face multifaceted challenges. For instance, individual dietary habits are often resistant to modification, and precise definitions of intake dosages and food sources are required. Furthermore, inflammatory biomarker levels are influenced by multiple confounding factors, necessitating longitudinal dynamic monitoring to evaluate intervention efficacy. However, frequent testing imposes additional burdens on patients.

In reverse MR, COPD also influenced MUFAs levels (IVW: OR 1.040, 95% CI 1.010–1.071, *p* = 0.008), MUFAs levels increased in COPD patients, which was consistent with the observational findings of Titz et al. [66]. Titz et al. identified a general increase in MUFAs regardless of smoking history in COPD patients. Serum lipidomic and weighted co‐expression network analysis of 160 subjects (40 with COPD) detected a significant divergence in lipid profiles when comparing smokers with COPD to healthy smokers. Key alterations included lower PUFA levels and higher MUFA levels [66]. They also identified a trend of decline in omega‐3 FAs [66], which did not show any significant value in our study (IVW: OR 0.994, *p* = 0.620). Another relevant study indicated elevated levels of MUFAs in smokers, who were at high risk for COPD [67]. One possible explanation for the increased proportion of MUFAs in the plasma of smokers is a change in endogenous FA desaturation, which may be caused by upregulation of stearoyl‐coenzyme A desaturase 1 (SCD1) [67]. Our MR study did not demonstrate the effects of COPD on DHA levels (IVW: OR 0.979, *p* = 0.159), LA level (IVW: OR 1.016, *p* = 0.289), and omega‐6 FA (IVW: OR 1.012, *p* = 0.477) and PUFA levels (IVW: OR 1.009, *p* = 0.555). Further investigations into the ratio of DHA levels to overall FA levels and the rate of MUFAs to overall FA levels may be meaningful. After all, the significance of this impact remains to be elucidated.

MR analysis has effectively demonstrated some causal risk factors for COPD. Kuryan et al. highlighted through the MR method that a history of smoking is significantly associated with increased susceptibility to COPD and its acute exacerbations [68]. Another MR study showed that air pollution, such as nitrogen oxides and particulate matter with diameter < 2.5 μm (PM_2.5_), is causally associated with an elevated risk of developing COPD. Normal lipid metabolism is essential for maintaining healthy lung function, and dysregulation of lipid metabolism contributes to the complex pathogenesis of COPD. Fatty acid β‐oxidation (FAO) may initially compensate to sustain ATP levels, but chronic exposure to cigarette smoke increases FAO, possibly exacerbating the accumulation of reactive oxygen species (ROS), mitochondrial damage, and cell death. This progress is modulated by the COPD susceptibility gene, family with sequence similarity 13 member A (FAM13A) [65]. Intervention strategies targeting key metabolic pathways such as FAO and the identification of regulatory genes may help alleviate oxidative stress, enhance mitochondrial function, and decrease apoptosis, thereby exerting beneficial effects on the treatment of COPD [69]. As a powerful tool for causal inference, MR studies provide unique and significant new insights into the complex etiology of disease development from a genetic perspective, effectively compensating for the limitations of traditional observational studies. This genetic research could groundbreaking insights into the origins of COPD, uncovering promising biomarkers and potential therapeutic targets. However, translating these discoveries into clinical practice faces significant hurdles, including genetic heterogeneity, the strong influence of environmental factors, and challenges in practical application.

There were several apparent advantages in our bidirectional MR analysis. First of all, our investigation was the initial study which examined the cause‐and‐effect association between UFAs and COPD by utilizing bidirectional two‐sample MR. Additionally, each GWSA data had a comparatively large sample scale, providing adequate statistical power. Thus, both exposure and outcome GWAS data were sourced from disparate databases, thereby minimizing the potential error from sample population [40]. In the last, we employed three methods in our sensitivity analysis: the heterogeneity analysis, the pleiotropy analysis, and the leave‐one‐out analysis, enhancing the robustness of our findings. The epidemiological significance of MR study is substantial, and its utilization is poised to expand further in the coming decades. With the elevating availability of genetic data and the development of novel methodologies, MR analysis will persist as an invaluable instrument for elucidating the causal connections between risk variables and disease outcomes [32].

However, our study also has limitations. Obviously, the European population from our study limited the broad applicability of our findings on a global scale. Additionally, we did not conduct subgroup analysis based on gender and smoking history, and the GWAS data from FinnGen did not describe the pathological phenotypes of COPD patients, such as chronic bronchitis or emphysema, as the results might vary across different populations. Furthermore, there was heterogeneity among SNPs for omega‐3 FA levels (IVW: *p* = 0.07; MR‐Egger: *p* = 0.019) and MUFA levels (IVW: *p* = 0.001; MR‐Egger: *p* = 0.003) through Cochran's *Q* test. Heterogeneity refers to differences among individuals in a study, which may stem from genotype, environmental factors, or other uncertainties. Lastly, conducting a multivariable Mendelian randomization (MVMR) was deemed necessary, as this approach helps to mitigate bias and confounding, thereby yielding more robust estimates of causal effects compared to single‐variable analysis [70]. To further define the biological pathways linking FAs to COPD pathogenesis, we plan future research involving cell‐based assays, tissue sequencing, in vivo models, and large‐scale clinical investigations.

## 5 Conclusion

To conclude, this research examined the bidirectional causal relationship between UFAs and COPD through MR within the European population for the first time. The findings supported UFAs as causal protective factors of COPD. The most important contribution of our study was that it could urge people to modify dietary nutrition, especially increasing the intake of PUFAs to prevent COPD. We hope the results could provide further knowledge regarding the development of reliable biomarkers and therapeutic targets in COPD. Moreover, RCT or real‐world studies are advocated to estimate causal effects between UFAs and COPD.

## Author Contributions

All authors read and approved the final manuscript. Study concept and design: Xiaoyong Shen, Xuelin Zhang, and Xiaojun Wang. Acquisition, analysis, or interpretation of data: Shuai Jiang, Lili Lu, Xunxia Zhu, and Xiaoyu Chen. Drafting of the manuscript: Shuai Jiang, Lili Lu, Xuanqi Liu, and Xuchao Gu.

## Funding

This research was supported by the Clinical Medical Research Project of Shanghai Science and Technology Commission (No. 21MC1930200).

## Consent

All authors read and approved this manuscript for publication.

## Conflicts of Interest

The authors declare no conflicts of interest.

## Supporting information


**Data S1:** Supporting Information.

**Figure S1:** This scatter plot shows the effects of unsaturated fatty acids (UFAs) on chronic obstructive pulmonary disease (COPD). (A) Association of docosahexaenoic acid levels and COPD. (B) Association of linoleic acid levels and COPD. (C) Association of omega‐3 fatty acid levels and COPD. (D) Association of omega‐6 fatty acid levels and COPD. (E) Association of polyunsaturated fatty acid levels and COPD. (F) Association of monounsaturated fatty acid levels and COPD.
**Figure S2:** This scatter plot shows the effects of COPD on UFAs. (A) Association of COPD and docosahexaenoic acid levels. (B) Association of COPD and linoleic acid levels. (C) Association of COPD and omega‐3 fatty acid levels. (D) Association of COPD and omega‐6 fatty acid levels. (E) Association of COPD and polyunsaturated fatty acid levels. (F) Association of COPD and monounsaturated fatty acid levels.

**Table S1:** Instrumental variables of UFAs and COPD.

## Data Availability

The GWAS datasets used in this study are available from the online repositories. The summary data for UFAs is available at https://www.ebi.ac.uk/gwas/; the summary data for UFAs are available at https://www.finngen.fi/en.
