# Supplementary material for: Association Between Unsaturated Fatty Acid Levels and Chronic Obstructive Pulmonary Disease: A Bidirectional Mendelian Randomization Study
Source: Clin Respir J. 2026 Mar 7;20(3):e70179. doi: 10.1111/crj.70179 (PMC12967058; doi:10.1111/crj.70179)
Supplement: Supplementary file 2 — Figure S1: This scatter plot shows the effects of unsaturated fatty acids (UFAs) on chronic obstructive pulmonary disease (COPD). (A) Association of docosahexaenoic acid levels and COPD. (B) Association of linoleic acid levels and COPD. (C) Association of omega‐3 fatty acid levels and COPD. (D) Association of omega‐6 fatty acid levels and COPD. (E) Association of polyunsaturated fatty acid levels and COPD. (F) Association of monounsaturated fatty acid levels and COPD. Figure S2: This scatter plot shows the effects of COPD on UFAs. (A) Association of COPD and docosahexaenoic acid levels. (B) Association of COPD and linoleic acid levels. (C) Association of COPD and omega‐3 fatty acid levels. (D) Association of COPD and omega‐6 fatty acid levels. (E) Association of COPD and polyunsaturated fatty acid levels. (F) Association of COPD and monounsaturated fatty acid levels. [file CRJ-20-e70179-s001.docx]

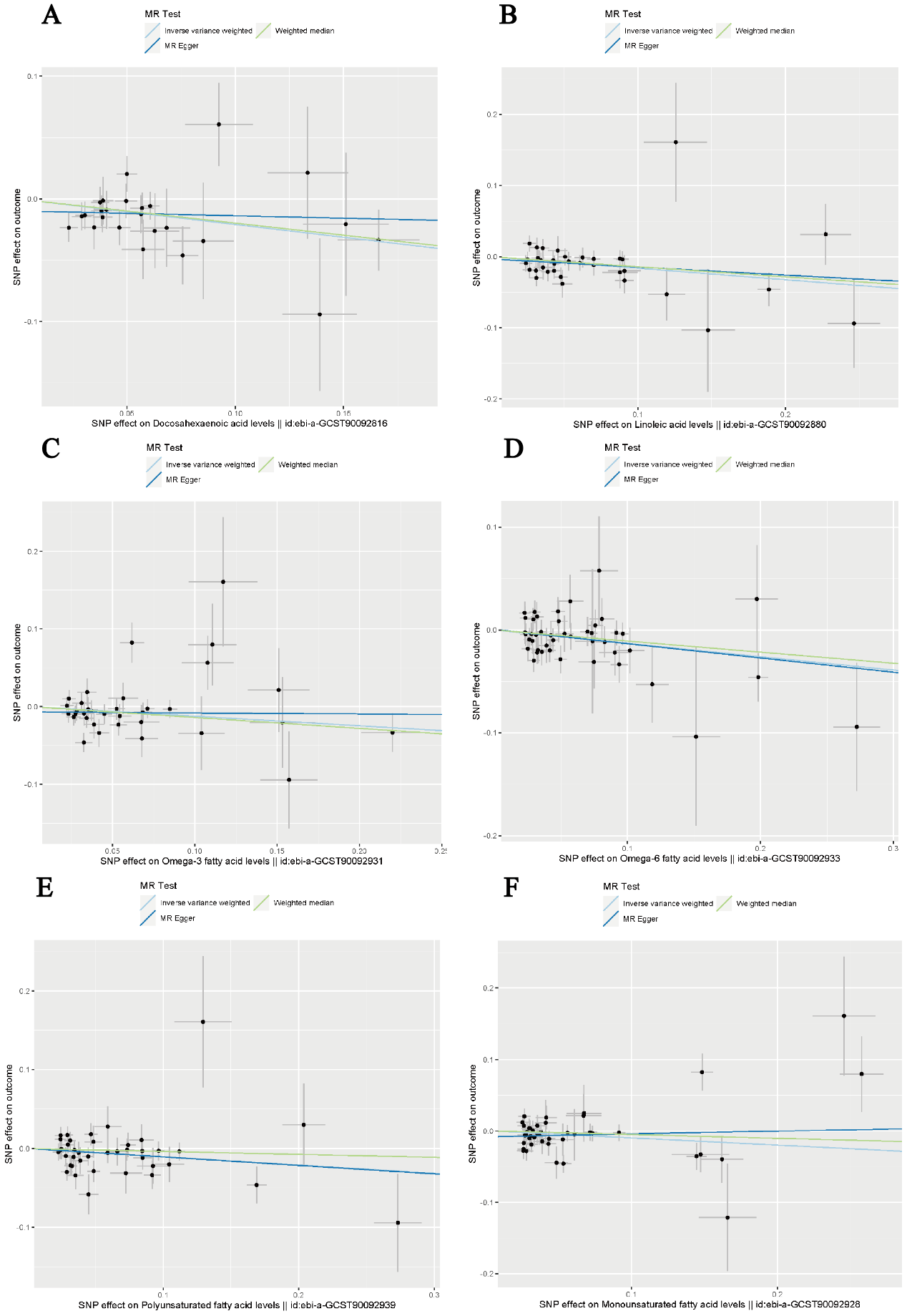


**Supplementary Figure 1:** This scatter plot shows the effects of unsaturated fatty acids (UFAs) on chronic obstructive pulmonary disease (COPD). **A:** Association of docosahexaenoic acid levels and COPD; **B:** Association of linoleic acid levels and COPD; **C:** Association of omega-3 fatty acid levels and COPD; **D:** Association of omega-6 fatty acid levels and COPD; **E:** Association of polyunsaturated fatty acid levels and COPD; **F:** Association of monounsaturated fatty acid levels and COPD.


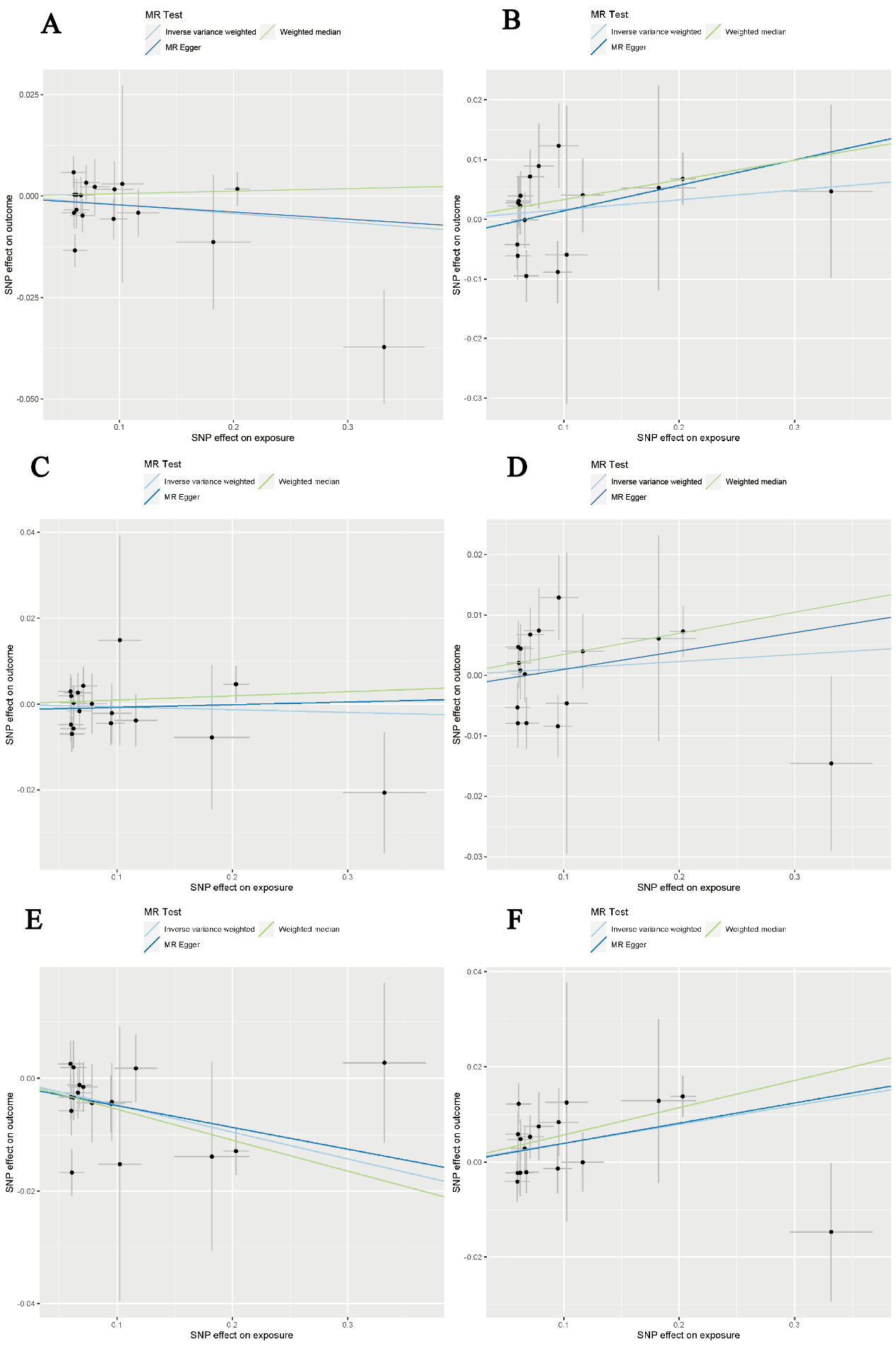


**Supplementary Figure 2:** This scatter plot shows the effects of COPD on UFAs. **A:** Association of COPD and docosahexaenoic acid levels; **B:** Association of COPD and linoleic acid levels; **C:** Association of COPD and omega-3 fatty acid levels; **D:** Association of COPD and omega-6 fatty acid levels; **E:** Association of COPD and polyunsaturated fatty acid levels; **F:** Association of COPD and monounsaturated fatty acid levels.
